# Uncommon Find: An Unusual 10 cm Bezold's Abscess Involving Dural and Mastoid Erosion

**DOI:** 10.7759/cureus.51298

**Published:** 2023-12-29

**Authors:** Jerry Cruz Rodriguez, Ana L Melero Pardo, Isabel Castellanos Castillo, Camila S Ríos de Choudens, Miguel Rivera

**Affiliations:** 1 Medicine, Universidad Central del Caribe, Bayamón, PRI; 2 Otolaryngology-Head and Neck Surgery, University of Puerto Rico School of Medicine, San Juan, PRI; 3 Otolaryngology-Head and Neck Surgery, University of South Florida, Tampa, USA

**Keywords:** bezold's abscess, mastoiditis, cervical neck abscess, otolaryngology-head and neck surgery, complications of acute otitis media

## Abstract

Bezold's abscess (BA) is a rare complication of otitis media that presents as a lateral neck abscess below the mastoid tip. BA incidence has recently decreased due to early diagnosis and prompt antibiotic intervention. We present a 42-year-old male with a complicated otitis media developing a 10 cm BA. Treatment of the lesion included surgical drainage and mastoidectomy, accompanied by intravenous (IV) broad-spectrum antibiotic administration. The patient experienced no adverse events during or after surgery and was placed on postoperative observation. However, on postoperative day (POD) 2, the patient left the hospital against medical advice and did not undergo further follow-up.

## Introduction

First described in 1881 by Friedrich Bezold, hence its name, Bezold's abscess (BA) is a complication of otitis media that manifests as lateral neck abscess below the mastoid tip with oozing of purulent secretions from the digastric groove and/or sternocleidomastoid muscles [1]. Before the antibiotic era, complications of otitis media (intracranial and extracranial) carried a significant risk of morbidity and mortality. However, due to the administration of antibiotics and the implementation of ventilation tubes, such complications have considerably decreased, making them relatively uncommon [2]. If left untreated, the secretions and bacteria can cause erosion of the mastoid process's medial wall and accumulate these materials in the digastric sulcus [3]. Further spread of the infection may lead to internal jugular vein thrombosis via penetration of the deep cervical fascia and acute mediastinitis via spread to the mediastinum [2]. Early diagnosis and management of a BA is accomplished by maintaining a high level of clinical suspicion and promptly utilizing radiologic imaging in the early stages [1].

Bacterial cultures have identified various sources of infection, out of which *Streptococcus pneumoniae *and *Streptococcus pyogenes* are the most frequently isolated microorganisms that lead to the development of a BA [2]. Despite its rarity, a BA can affect people of all ages. Pneumatization of mastoid cells tends to occur later in life. Therefore, most cases are reported in the adult population due to thinning of the mastoid bone, favoring erosion in this population [3]. Furthermore, it tends to affect patients with limited access to healthcare due to a lack of early disease management and antibiotic therapy and in an immunocompromised state [2].

## Case presentation

**Figure 1:**
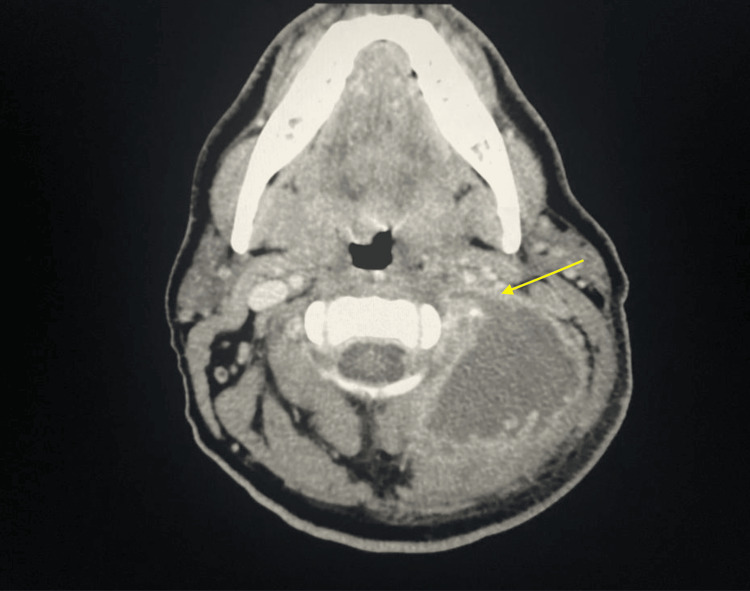
Non-contrast axial neck soft tissue CT scan demonstrating occlusion of neck vessels

**Figure 2:**
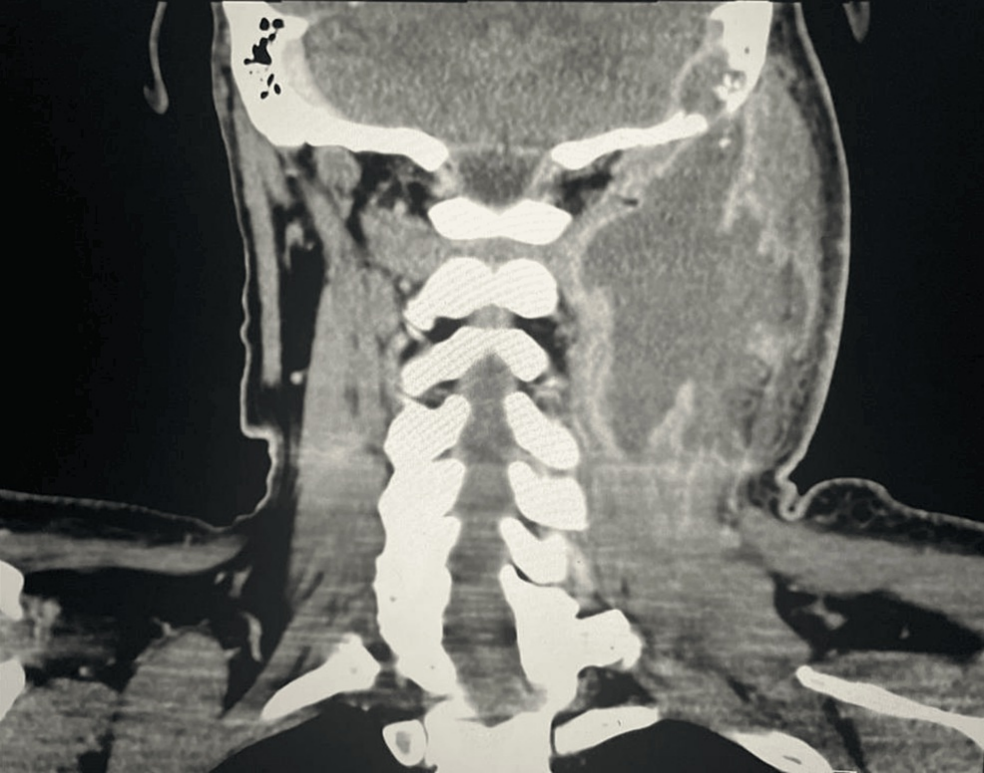
Non-contrast coronal CT scan of the neck revealing well-defined BA measuring 10 cm in diameter BA: Bezold's abscess

**Figure 3:**
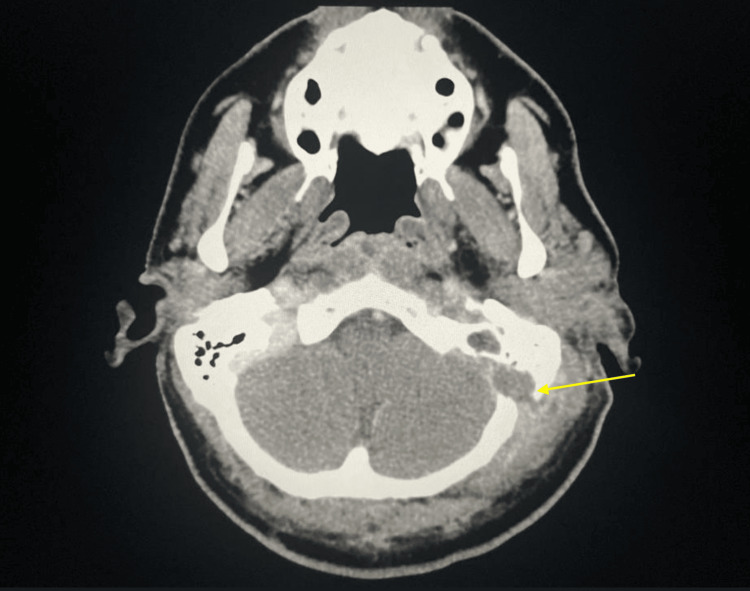
Non-contrast axial view of the skull revealing erosion of mastoid process and dural enhancement

A 42-year-old male presented to the emergency room complaining of severe left ear pain, headaches, left neck swelling, and a febrile episode of 101.2°F. The patient reported experiencing left ear pain that started several weeks prior to arrival at the emergency room. Upon examination, pain was accompanied with soreness at the mastoid tip and an enlarging neck mass which the patient noticed a few weeks after ear pain. The patient described the mass as bothersome and tender to touch. Physical examination revealed purulent otorrhea in his left ear and tenderness with fluctuant swelling arising from the left mastoid process. Table 1 illustrates the patient's laboratory findings, showcasing an elevated white blood cell (WBC) count of 14.4×10^9^/L (differential count reveals neutrophils at 10.6×10^9^/L and monocytes at 1.2×10^9^/L). Other notable findings include hyperglycemia, with a blood glucose level of 409 mg/dL and mild anemia with a hemoglobin (HGB) level of 12.4 g/dL. Subsequently, a neck CT scan with intravenous (IV) contrast revealed a 10 cm rim-enhancing abscess multi-lobulated loculation extending superiorly from the eroded skull base to posteroinferiorly into the supraclavicular region of the neck. Additionally, there was evidence of dural enhancement attributed to the erosion of the mastoid process caused by the abscess. Due to its size, the abscess compressed the left jugular vein and the transverse and the sigmoid sinuses. The CT scan's findings, Figures 1-3, were consistent with a BA due to its extent and affected structures. Due to BA's rare, acute, and progressive nature, coupled with constitutional symptoms and secondary manifestations due to mass compression, BA might be mistaken for head and neck malignancies, tuberculosis, suppurative lymphadenopathy, or cystic conditions upon initial examination [4].

**Table 1:** Patient laboratory evaluation CBC: complete blood count; H: high; L: low; Abs: absolute; WBC: white blood cell; RBC: red blood cell; HGB: hemoglobin; HCT: hematocrit; MCV: mean corpuscular volume; MCH: mean corpuscular hemoglobin; MCHC: mean corpuscular hemoglobin concentration; RDW: red cell distribution width; PLT: platelets; CMP: comprehensive metabolic panel

Test	Observed value	Reference range
CBC		
WBC	14.4 (H)	4.0-11.0x10^3^/µ
RBC	4.15	4.0-5.0x10^6^/µL
HGB	12.4 (L)	13.5-17.5 g/dL
HCT	38.2 (L)	41-50%
MCV	92	80-100 um
MCH	29.8	25-35 pg/cell
MCHC	32.4	31-36% Hb/cell
RDW	12.2	11.8-14.5%
PLT	320x10^3^/µL	150-450x10^3^/µL
Lymphocytes	16.5% (L)	25-33%
Monocytes	0.083	3-7%
Eosinophils	0.009	1-3%
Basophils	0.005	0-0.75%
Neutrophils, Abs	10.6 (H)	1.50-7.0x10^3^/µL
Lymphocytes, Abs	12.4	1.0-3.7x10^3^/µL
Monocytes, Abs	1.2 (H)	0-0.70x10^3^/µL
Eosinophils, Abs	0.1	0-0.40x10^3^/µL
Basophils, Abs	0.1	0-0.10x10^3^/µL
CMP		
Glucose	409 (H)	70-100 mg/dL
Sodium	137	135-145 mmol/L
Potassium	4	3.5-5.0 mmol/L
Chloride	101	96-106 mmol/L
Carbon dioxide	25	23-29 mmol/L
Blood urea nitrogen	7	7-20 mg/dL
Creatinine	0.72	0.6-1.3 mg/dL
Calcium	8.4 (L)	8.6-10.6 mg/dL

Surgical management was done with left lateral neck exploration, which incorporated a dissection of the sternocleidomastoid into the suprasternal space and a mastoidectomy. Furthermore, the wound was cultivated, and the area was irrigated with clindamycin. Cultures were remarkable for *S. pneumoniae*. Following neck incision, erosion of the posterior fossa, sigmoid sinuses, and mastoid cavity was discovered during the intervention; however, there were no signs of cholesteatoma. There was no need to place a tympanostomy tube because of a sizable tympanic membrane perforation. The patient was transferred to the intensive care unit, where he was closely monitored with IV broad-spectrum antibiotics and ciprofloxacin/dexamethasone ear drops. Administration of antibiotics, mastoidectomy, debridement, and drainage are the mainstay treatment for BA as it often results in the complete resolution of the abscess and to prevent additional complications. The following day, the patient was stable, fully conscious, tolerating oral diet, and reporting minimal pain. On postoperative day (POD) 2, the patient left the hospital against advice with no subsequent follow-up.

## Discussion

In this case report, we highlight a BA, a rare suppurative extracranial complication of acute otitis media, with a diameter of 10 cm with partial compression of the left jugular veins as well as the transverse and sigmoid sinuses. Between 2000 and 2020, 28 cases of BA were published on PubMed [5]. The majority of the reported cases measured 6 cm or smaller, and only one case reported an abscess diameter of 10 cm, highlighting the rarity of this size [5]. Depending on the extent of BA, multiple head and neck structures might be compressed, leading to further complications in the patient, making a 10 cm abscess highly problematic. Thorough evaluation of patient history and symptomatology are crucial in patients presenting with otitis media and mastoiditis as BA often develops behind the neck muscles, presenting a challenge for detection through a neck examination [3,5]. Common indicators of BA include progressive fever, neck swelling, facial paralysis, neck pain, otorrhea, hearing loss, and ear pain [6]. Laboratory workup, along with physical exam and history, is often supportive in the diagnosis of BA, as there is usually elevated leukocyte count and erythrocyte sedimentation rate [5]. CT scan is the imaging modality of choice in patients with BA as it allows visualization of the extent of the abscess and involvement of the mastoid process and adjacent affected structures [3]. Furthermore, CT scan allows the detection of mastoid process origin in cases of abscess formation in the perivertebral and/or posterior cervical space [3].

The classical appearance of mastoid erosion and dural enhancement on imaging studies in this patient played a crucial role in the prompt decision to perform urgent surgical drainage and mastoidectomy. This step is deemed vital for the proper management of BA, which allowed the removal of infected tissue and osteolytic bone. Before the advent of antibiotics and modern imaging techniques, BA could persist for an extended period, potentially resulting in severe complications. These complications include compression of head and neck veins, thrombosis, and extension into the skull base or the vertebrae, leading to brain and spinal cord compression, which could ultimately result in death [3]. Currently, treatment for BA is determined based on the severity and extent of the abscess. Besides the administration of antibiotics, surgical mastoidectomy, debridement, and drainage are often necessary for the resolution of the abscess and to prevent additional complications. Of the 28 patients with data regarding treatment, current measures reduce the possibility of further expansion of BA, leading to associated complications.Streptococci are the most common causative organisms of BA reported in the literature [5].

BA is considered very rare due to the current treatment of otitis media, especially as soon as bothersome symptoms appear. However, the case presented in this report highlights how lack of access to medical care and antibiotics leads to the appearance of this complication. This care report highlights a rare 10 cm BA with dural and mastoid erosion. The abscess was successfully removed via surgical drainage and mastoidectomy. The patient showed no immediate postoperative complications during his hospital stay after receiving standard care. No further data is available after POD2.

## Conclusions

BA is a rare complication of otitis media that presents as a lateral neck abscess and mastoid process erosion. Diagnosing and treating BA is accomplished by maintaining a high level of clinical suspicion and promptly utilizing radiologic imaging to assess size and affected structures. In this case report, we present a rare 10 cm BA that resulted from an untreated acute otitis media. The patient underwent surgical drainage and mastoidectomy, along with IV broad-spectrum antibiotics. Today, BA are rare due to the early management of acute otitis media preventing further complications. However, a high clinical suspicion must be present in a patient with a history of otitis media, mastoiditis, and neck swelling, as exemplified by the scenario of this case report, so that adequate and timely treatment is rendered to reduce additional deterioration.

## References

[1] Lin YH, Lin MY (2015). Bezold abscess. Ear Nose Throat J.

[2] Govea-Camacho LH, Pérez-Ramírez R, Cornejo-Suárez A, Fierro-Rizo R, Jiménez-Sala CJ, Rosales-Orozco CS (2016). Diagnosis and treatment of the complications of otitis media in adults. Case series and literature review [Article in Spanish]. Cir Cir.

[3] Castillo M, Albernaz VS, Mukherji SK, Smith MM, Weissman JL (1998). Imaging of Bezold's abscess. AJR Am J Roentgenol.

[4] Winters R, Hogan CJ, Lepore ML, Geiger Z (2023). Bezold Abscess. StatPearls [Internet].

[5] Alkhaldi AS, Alwabili M, Albilasi T, Almuhanna K (2022). Bezold's abscess: a case report and review of cases over 20 years. Cureus.

[6] Marioni G, de Filippis C, Tregnaghi A, Marchese-Ragona R, Staffieri A (2001). Bezold's abscess in children: case report and review of the literature. Int J Pediatr Otorhinolaryngol.

